# 40 years of biannual family medicine research meetings – The European General Practice Research Network (EGPRN)

**DOI:** 10.3109/02813432.2013.847594

**Published:** 2013-12

**Authors:** Nicola Buono, Hans Thulesius, Ferdinando Petrazzuoli, Tiny Van Merode, Tuomas Koskela, Jean-Yves Le Reste, Hanny Prick, Jean Karl Soler

**Affiliations:** ^1^European General Practice Research Network Council and Executive Board; ^2^EGPRN Office, Department of Family Medicine, University of Maastricht, The Netherlands

**Keywords:** Europe, family medicine, general practice, research, research network, EGPRN, national representative, membership

## Abstract

**Objective:**

To document family medicine research in the 25 EGPRN member countries in 2010.

**Design:**

Semi-structured survey with open-ended questions.

**Setting:**

Academic family medicine in 23 European countries, Israel, and Turkey.

**Subjects:**

25 EGPRN national representatives.

**Main outcome measures:**

Demographics of the general population and family medicine. Assessments, opinions, and suggestions.

**Results:**

EGPRN has represented family medicine for almost half a billion people and > 300 000 general practitioners (GPs). Turkey had the largest number of family medicine departments and highest density of GPs, 2.1/1000 people, Belgium had 1.7, Austria 1.6, and France 1.5. Lowest GP density was reported from Israel 0.17, Greece 0.18, and Slovenia 0.4 GPs per 1000 people. Family medicine research networks were reported by 22 of 25 and undergraduate family medicine research education in 20 of the 25 member countries, and in 10 countries students were required to do research projects. Postgraduate family medicine research was reported by 18 of the member countries. Open-ended responses showed that EGPRN meetings promoted stimulating and interesting research questions such as comparative studies of chronic pain management, sleep disorders, elderly care, healthy lifestyle promotion, mental health, clinical competence, and appropriateness of specialist referrals. Many respondents reported a lack of interest in family medicine research related to poor incentives and low family medicine status in general and among medical students in particular. It was suggested that EGPRN exert political lobbying for family medicine research.

**Conclusion:**

Since 1974, EGPRN organizes biannual conferences that unite and promote primary care practice, clinical research and academic family medicine in 25 member countries.

- The European General Practice Research Network (EGPRN) is a 40-year-old organization with 25 member countries representing almost 500 million people served by > 300 000 GPs.
- EGPRN arranges biannual meetings to promote and unite primary care practice, family medicine clinical research, and academic family medicine.
- There is a large variation between countries in academic departments and research networks and EGPRN serves an important role in harmonizing this diversity.

## Biannual European Research Network in Family Medicine since 1974

Family medicine has matured as an academic and scientific discipline [1] and the European General Practice Research Network (EGPRN, http://www.egprn.org) has organized biannual family medicine research meetings in Europe, to develop and showcase family medicine research since 1974. During these 40 years EGPRN, which until 2003 was called EGPRW – the W meaning Workshop – has offered research courses, workshops on methodology, published position papers in various European journals, mentored individual researchers, supported research presentations at WONCA Europe conferences, supported international collaborative family medicine research projects, and developed a Research Agenda for European family medicine research [2]. EGPRN is part of the research stream within WONCA (World Organization of Family Doctors) Europe and as a result of this the EGPRN International Family Medicine Research Course was developed [3].

EGPRN meetings are characterized by an intimacy due to their small size in terms of numbers of participants. The limited size allows for more feedback and discussions during and after presentations than in traditional conferences. This tradition of feedback discussions relates to the previous EGPRW name of the association.

## Research in Family Medicine for half a billion people and > 300 000 GPs!

EGPRN National Representatives were key informants for this report with the aim to document family medicine research capacity in 25 EGPRN member countries including Israel and Turkey in a survey ending in 2010. A total of 25 EGPRN national representatives reported demographic data and described research capacity in their home countries in semi-structured questionnaires with open-ended response options. Demographic characteristics of family medicine and population numbers in EGPRN member countries are shown in Table I. EGPRN covers family medicine in a population of almost half a billion people with over 300 000 general practitioners (GPs). Turkey reported the largest number of family medicine departments and also the highest density of GPs with 2.1/1000 people followed by Belgium 1.7, Austria 1.6, and France 1.5. The lowest GP density was reported from Israel 0.17, followed by Greece 0.18, and Slovenia with 0.4 GPs per 1000 people.

**Table I.** Demographic characteristics of family medicine and population numbers in the 25 EGPRN member countries 2010.

Countries	No. of GPs	No. of inhabitants (thousands)	No. of EGPRN members	No. of GPs per 1000 inhabitants	No. of departments of family medicine	Number of FM departments per million inhabitants
Austria	11 716	7000	1	1.60	3	0,43
Belgium	18 217	10 414	19	1.70	5	0,48
Bulgaria	5402	7358	4	0.73	5	0,68
Croatia	2350	4437	12	0.50	1	0,23
Denmark	3400	5000	1	0.68	3	0,60
Estonia	1000	1340	1	0.74	1	0,75
Finland	3500	5300	1	0.70	5	0,94
France	94 909	64 658	28	1.46	30	0,46
Germany	58 095	82 369	12	0.70	35	0,42
Greece	1800	1000	7	0.18	1	1,00
Hungary	6381	10160	4	0.63	4	0,39
Ireland	2500	4239	6	0.59	3	0,71
Israel	1300	7509	5	0.17	4	0,53
Italy	45 000	56 995	8	0.90	–	
Netherlands	8500	16 000	15	0.50	8	0,50
Latvia	1283	2254	2	0.60	–	
Lithuania	–	3300	1	–	–	
Malta	325	400	1	0.81	1	2,50
Norway	4000	4858	4	0.82	4	0,82
Portugal	5055	10 356	2	0.50	6	0,58
Slovenia	872	2032	2	0.40	2	0,98
Sweden	4800	9340	1	0.51	7	0,75
Switzerland	6408	7800	2	0.61	–	
Turkey	15 000	72 000	7	2.10	54	0,75
United Kingdom	37 500^1^	63 000^1^	5	0.60^1^	–	
Total	339 313	458 119	150	Mean 0.74	182	Mean 0,40

Note: ^1^Data from a 2012 study.

Although family medicine research is often considered as less important than secondary care research [4] most countries have research capacities in many academic departments and research networks in family medicine were reported in 22 out of 25 EGPRN member countries. Undergraduate education in family medicine research was indicated in 20 of the 25 (80%) countries, and in 10 (40%) of the countries students were required to do a research project. Postgraduate family medicine research was reported by 18 (72%) of the countries and was compulsory in 10 (44%). The national representative reports included open-ended questions on membership issues, EGPRN meetings, and potential new research topics and showed that EGPRN meetings promoted stimulating and interesting research questions. It was suggested that the EGPRN exert more political lobbying for family medicine research in European policy-making. EGPRN members expressed interest in research themes such as: comparative studies of chronic pain management, sleep disorders, elderly care, healthy lifestyle promotion, improving mental health, clinical competence, and appropriateness of specialist referrals.

## Academic family medicine: weak but growing

Many national representatives reported a general lack of interest in family medicine research in their countries, mainly related to poor incentives and low family medicine status in general and among medical students in particular [5,6]. Even if academic family medicine infrastructure seemed well developed in many countries there were differences when considering numbers and types of research projects, and undergraduate and postgraduate education. The career tracks for becoming a family medicine professor were also different throughout Europe [7].

This study comes from a broad range of countries covered by EGPRN national representative reports that are generally well informed regarding the family medicine research situation in their countries. Hence, their reflections are valuable to comprehend family medicine research in Europe, Israel, and Turkey today. Family medicine research is growing in the majority of EGPRN member countries, but barriers to further development exist and cooperation is therefore a must. In Northern Europe family medicine research collaboration in the Forum Balticum workshops between the Baltic and Scandinavian countries has been fruitful with EGPRN members engaged in that work [8].

## EGPRN challenged by other conferences

EGPRN is a 40-year-old research network involving people from countries with a population of almost 500 million people and > 300 000 GPs, including Turkey and Israel as European neighbour members. Medical research has traditionally been based in academic centres, and the findings are frequently not applicable in community primary care settings. The result is a large gap between the possible and the practical in delivering high-quality primary care. EGPRN is a laboratory for primary care clinical research and a vehicle for uniting primary care practice and family medicine clinical research in the European Community. Academic family medicine is still weak in most member countries, which indicates that EGPRN probably should do more EU lobbying to promote family medicine and family medicine research development. A stronger relationship with the European Community could enhance the availability of funding resources for such development and also boost network activities aimed at supporting family medicine research. Larger disease-specific conferences and the conferences of WONCA Europe Specific Interest Groups (WESIGs) compete with EGPRN meetings for European researchers. This competition is a challenge for EGPRN, which should define a better role for itself and communicate this role effectively to family medicine researchers looking for open and creative international collaboration.
